# Patient-specific simulation for tracheobronchial reconstruction procedures using 3-dimensional operable models: A proof-of-concept study

**DOI:** 10.1016/j.xjtc.2022.02.023

**Published:** 2022-02-21

**Authors:** Kohei Hashimoto, Kenshiro Omura, Naoya Iwamoto, Junji Ichinose, Yosuke Matsuura, Masayuki Nakao, Mingyon Mun

**Affiliations:** Department of Thoracic Surgical Oncology, Cancer Institute Hospital, Japanese Foundation for Cancer Research, Tokyo, Japan

**Figure:**
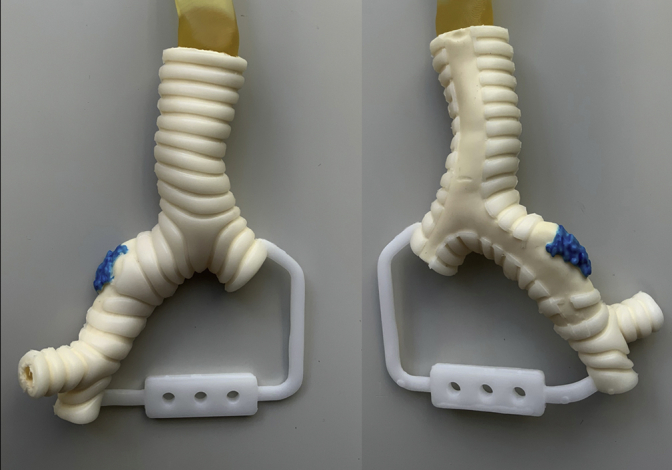
An operable 3D airway model for patient-specific simulation based on computed tomography.

**Central Message**

Operable 3D printed airway models with precise human anatomy, including invasive lesions, demonstrate promise for practical, patient-specific surgical simulation for tracheobronchial reconstruction.

In the Cone of Experience claimed by Edgar Dale in 1946, simulated activity was considered the second-best learning experience after actual activity.^1^ This is still valid in modern surgical practice where both patient safety and learning experience need to be protected. Surgical simulation may be particularly beneficial for rare, complex, and high-risk procedures such as tracheobronchial reconstruction. Operable airway models using preoperative computed tomography (CT) data were developed to assist in the patient-specific simulation of tracheobronchial reconstruction procedures.

## Methods

**Figure 1:**
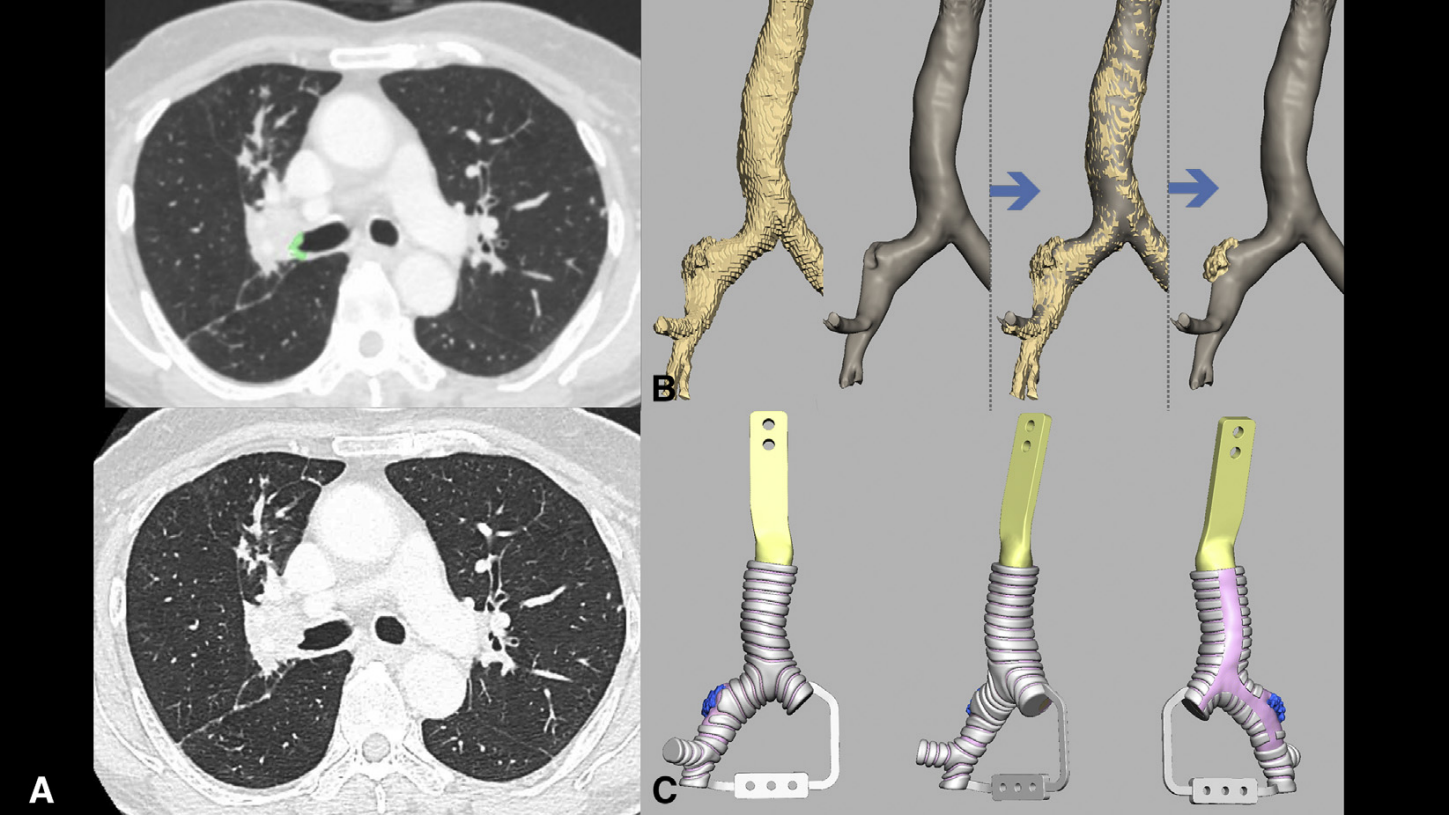
The sequence of the creation of the model (case 1: *right upper* sleeve lobectomy). A, *Upper*: The invasive area on the central airway was annotated (*green*) on preoperative chest computed tomography (CT) images. *Lower*: The same slice of the chest CT without annotation (raw CT data). B, The demarcation of the invasive lesion in the central airway by subtracting raw CT data from annotated CT data. C, The design of a 3-dimensional airway model using data obtained from chest CT. Note that the *pink-area* (connective tissue) is separately recognized from the *white area* (cartilage).

This retrospective study included 3 patients with non–small cell lung cancers who underwent sleeve lobectomies: right upper sleeve lobectomy (case 1, a 76-year-old man), right middle and lower sleeve lobectomy (case 2, a 60-year-old woman), and left lower and lingular segment extended sleeve lobectomy (case 3, a 59-year-old man). The invasive lesion in the central airway was annotated (Synapse Vincent; Fujifilm) in each preoperative enhanced chest CT (slice thickness, 1.25 mm) image (Figure 1, *A*). The Digital Imaging and Communications in Medicine data were converted to 3-dimensional (3D) data (OsiriX MD version 12.0; Pixmeo). The invasive lesion was demarcated by subtracting the CT data from the annotated CT data (Figure 1, *B*). The airway structure was determined, and the cartilage was distinguished from other connective tissues (Geomagic Freeform; 3D Systems) (Figure 1, *C*). The data were then converted into the Standard Triangle Language format for 3D printing using the Geomagic Freeform software. The hard plastic models of the cartilage and other tissues were separately 3D-printed (SCS-8100; Sony Manufacturing Systems) to serve as frameworks for the creation of silicone molds. Two urethan materials mimicking the cartilage (Hapla Pudding Gel-PL00; Polysis) and the remaining connective tissue, including the invasive lesion (Adapt, RU-843A-N80; Nisshin Resin) were poured into the molds, while the 2 parts were combined using a vacuum casting method (CrossMedical). Gauze strips were integrated into the connective-tissue part to strengthen the cut-resistance. The invasive lesions were highlighted in blue. Each model was evaluated by the surgeon of each operation. This study was approved by our institutional ethical review board (No. 2020-GA-1334) on May 28, 2021, and consent was waived because of its retrospective nature.

## Results

**Figure 2:**
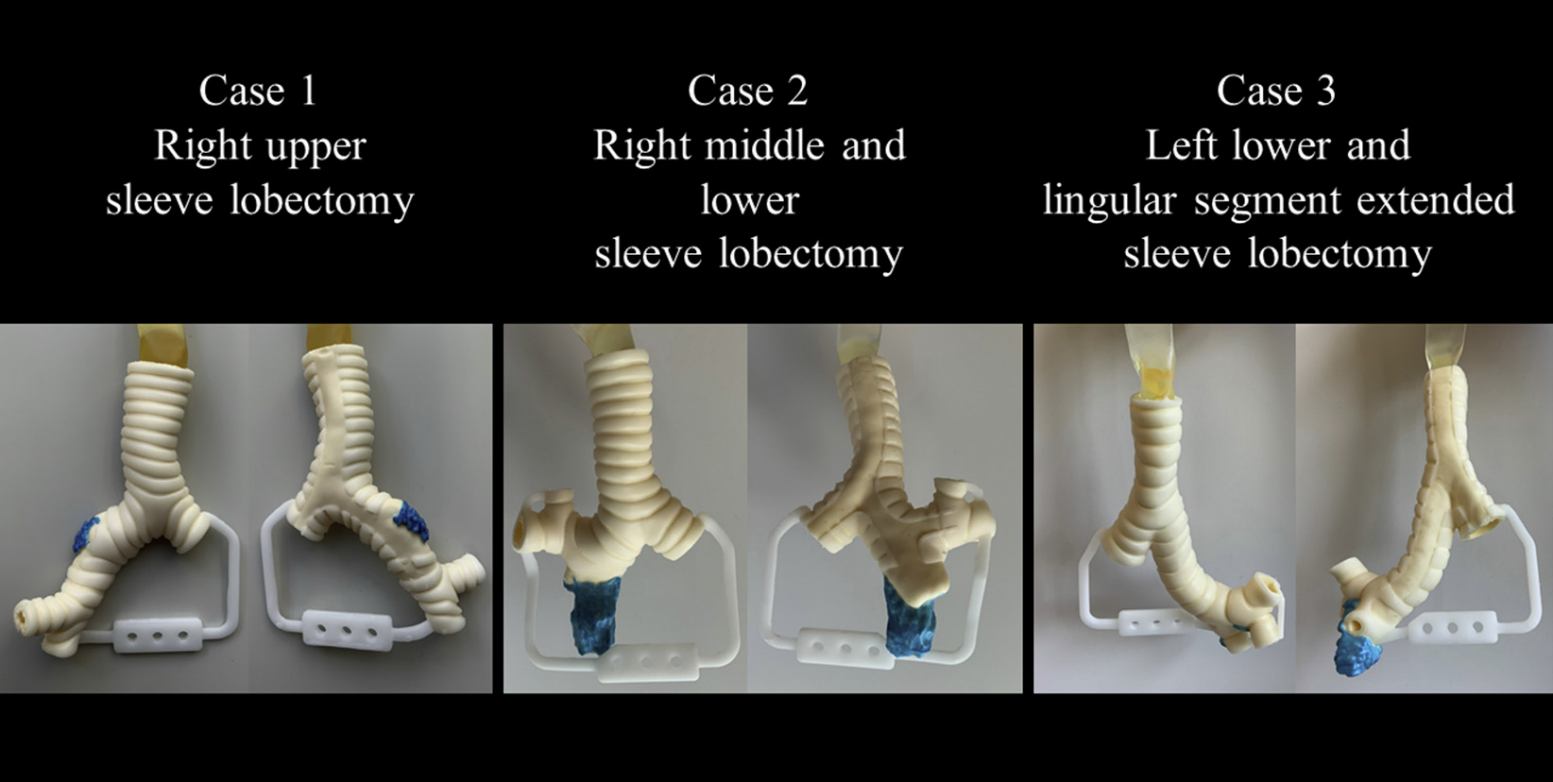
A 3-dimensional airway model consisting of multiple urethan materials representing the cartilage and the remaining connective tissue, including the invasive lesion in the central airway. Note that the invasive lesion is highlighted in *blue color* on the outer surface.

Three models, including invasive lesions, were successfully created (Figure 2). Three surgeons successfully reproduced the surgical procedures in the 3D airway models (Video 1). The models were rated based on how closely these reproduced actual surgeries (Likert scale where 1 = poor to 5 = excellent). The median scores were acceptable: anatomical reproducibility, 5 (range, 4-5); disease reproducibility, 4 (range, 4-5); surgical exposure, 4 (range, 3-5); rigidity, 3 (range, 2-4); elasticity, 4 (range, 3-5); resistance to needles, 3 (range, 2-4); and resistance to tying, 3 (range, 3-4). The time from initial planning chest CT (re-evaluation CT was performed, when necessary, which did not change the surgical plan) to actual surgeries in cases 1, 2, and 3 were 25, 36, and 27 days, respectively. The time from data extraction to receiving of the model was 31, 17, and 17 days, respectively in these retrospective settings. The authors (thoracic surgeons) needed approximately an hour to depict disease lesions using the software, extract data, and examine the 3D design of the model before manufacturing each model.

## Discussion

In this retrospective study, the feasibility of creating an interactive 3D airway model for a patient-specific tracheobronchial simulation was proven. Theoretically, any type of invaded airway can be created using this methodology. The cases included in this study are usually manageable without simulation by experienced surgeons. However, simulation may benefit junior surgeons and even experienced surgeons when they encounter highly complex reconstruction, such as carinal reconstruction.^2^^,^^3^ In the field of thoracic surgery, 3D-printed models have been proposed for the purpose of surgical simulation by appreciating real-size anatomy.^4^ However, our airway model is the first interactive patient-specific model for tracheobronchial surgery to our knowledge. Compared with existing simulation models based on animals or cadavers, we believe that our model has an advantage due to its portability and better anatomical clarity, including target diseases.

We required 31 days for creating a model for case 1 because it involved a trial-and-error process. Based on the knowledge obtained from the first model, the latter 2 models, for cases 2 and 3, were successfully made within 3 weeks. With this model, we plan to create a program of patient-specific simulation for tracheobronchial surgery.

## Conclusions

3D-printed operable airway models representing precise airway anatomy, including invasive lesions, were successfully created based on preoperative CT data, demonstrating promise for prospective patient-specific simulation for tracheobronchial reconstruction.

## Supplementary Data

Video 1 The demonstration of comparison between sleeve lobectomies reproduced using the airway model and the actual surgeries (cases 2 and 3). Video available at: https://www.jtcvs.org/article/S2666-2507(22)00131-6/fulltext.
