# Supplementary figures and images for: Patient-specific simulation for tracheobronchial reconstruction procedures using 3-dimensional operable models: A proof-of-concept study
Source: JTCVS Tech. 2022 Feb 21;14:138–40. doi: 10.1016/j.xjtc.2022.02.023 (PMC9366179; doi:10.1016/j.xjtc.2022.02.023)

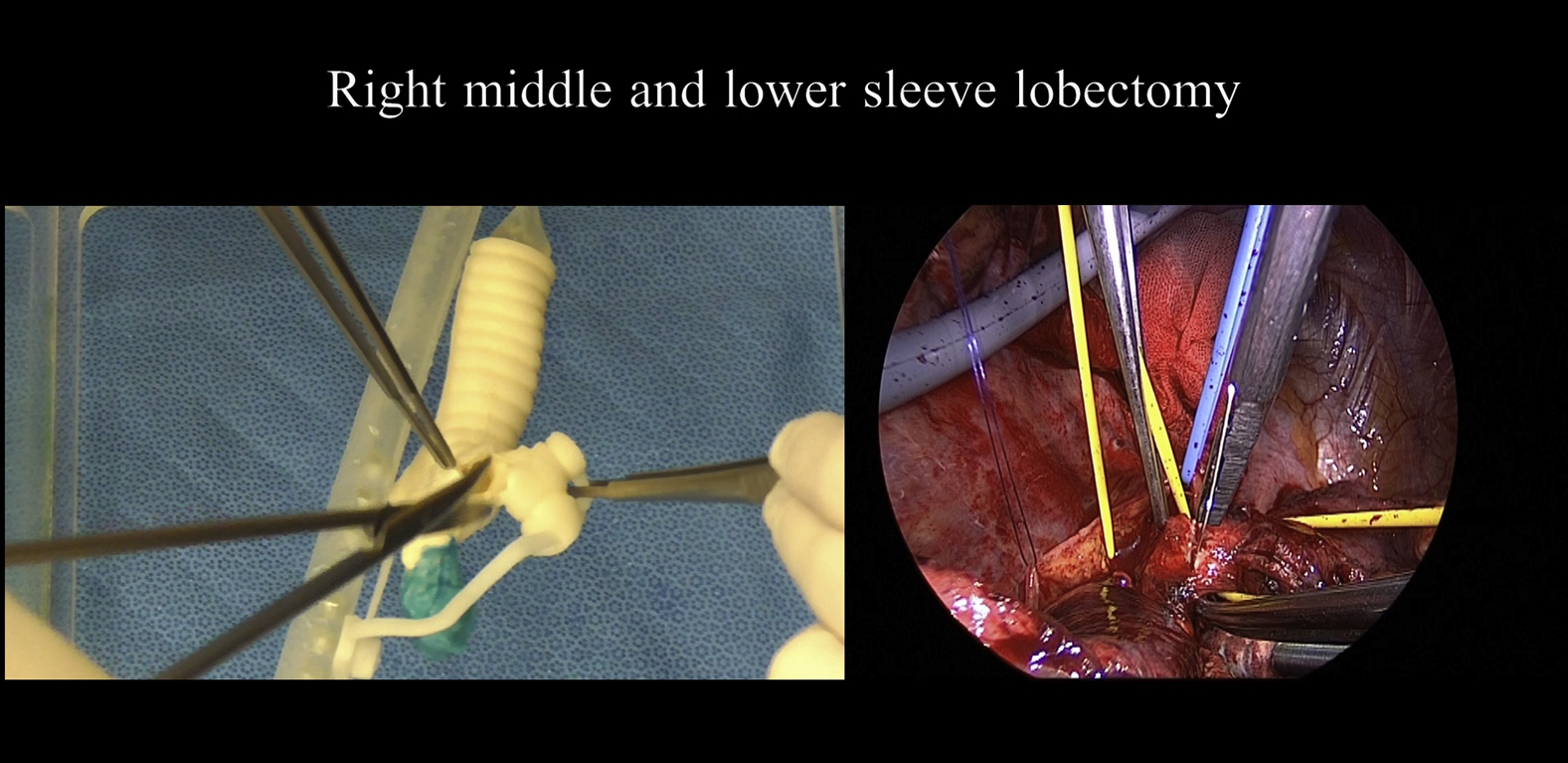

Supplement: Video 1 — The demonstration of comparison between sleeve lobectomies reproduced using the airway model and the actual surgeries (cases 2 and 3). Video available at: https://www.jtcvs.org/article/S2666-2507(22)00131-6/fulltext. [file fx2.jpg]
